# Adjacent Segment Disease (ASD) in Incidental Segmental Fused Vertebra and Comparison With the Effect of Stabilization Systems on ASD

**DOI:** 10.7759/cureus.18647

**Published:** 2021-10-10

**Authors:** Mehdi Hekimoğlu, Ahmet Başak, Atilla Yılmaz, Hakan Yıldırım, Ahmet Levent Aydın, Kursat Karadag, Ali Fahir Özer

**Affiliations:** 1 Neurosurgery, American Hospital, Istanbul, TUR; 2 Neurosurgery, Okan University, Istanbul, TUR; 3 Radiology, American Hospital, Istanbul, TUR; 4 Neurosurgery, Koc University School of Medicine, Istanbul, TUR; 5 Neurosurgery, Atatürk University, Erzurum, TUR

**Keywords:** incidental fusion, dynamic stabilization, fusion surgery, adjacent segment disease, spine stabilization

## Abstract

Objective

Adjacent segment disease is a controversial process after spine stabilization. The two important factors discussed are natural aging and hypermobility in incidental segmental fusion anomalies; patients have two or more fused vertebrae from birth, which are the results of spinal movement restriction due to the fusion of some spinal units. This article’s main purpose is to determine the degree of relationship of hypermobility and the aging process in the deterioration of the disks adjacent to fusion.

Methods

In this study, the degenerative process developed by hypermobility in the adjacent segment due to incidental segmental fusion was evaluated. The MRI images of 52 adjacent and nonadjacent disks of 45 patients in total were analyzed according to the Pfirrmann grading systems. The average Pfirrmann rating of the disks just above and below the fused segment and the distant first, second, and third non-neighboring levels were evaluated and calculated, respectively.

Results

The highest rate of incidental fusion is determined on the cervical area with 51.9%, followed by the thoracal area with 32.7%, and the lumbar area with 15.4%. Damage to the adjacent segment disks in cases with incidental fusion can still be seen at any age, with fusion, indicating that the hypermobility effect plays a more prominent role. The evidence of hypermobility without aging is that the segments adjacent to fusion undergo more degeneration than the distant disks.

Conclusion

Adjacent segment disease is under the influence of many factors. Our findings suggest that its incidence is increasing with the pathological processes initiated by hypermobility. It seems that, at least, it carries equal importance as compared to age. Fusion surgeries damage the adjacent segments under the influence of the passage of time beyond the physiological aging of the patient.

## Introduction

Adjacent segment disease (ASD) is a challenging condition, which may occur following spinal stabilization surgeries and affect outcomes. There is no common consensus about the main causes of ASD, although the etiology is multifactorial. Criteria such as genetic predispositions, surgical techniques, body mass index, preoperative mapping, and the peroperative use of motion-preserving systems will obviously help the proportional distribution of the loads and play a significant role in the ASD occurrence rate.

The proper selection of instrumentation techniques, the length and diameter of screws, the degree of insertion, and fusion length are important. Deciding at the point to start and to end the instrumentation materials is crucial to avoid overloading of a particular level by creating hypermobility. Awareness of the human spine's physiologic mobile segment can prevent malalignment, hypermobility, and ASD. Attention to preserving the normal sagittal balance peroperatively will reduce the risk of malalignment, consequently preventing overloading on the disk materials and ASD [1].

The question is, does adjacent segment degeneration occur in a natural human aging process, or is it the result of hypermobility due to the use of fusion stabilization techniques? Dynamic stabilization systems can overcome these problems.

Fusion surgeries are common procedures applied in spine surgery. In some cases, the development of ASD is reported following fusion systems; however, the same reports are seen in dynamic system techniques. Therefore, it encourages us to find a way to explain this controversy.

Incidental fusion is a condition wherein one or more vertebrae columns are fused together. It is part of some incidental syndromes, such as Klippel Feil syndrome, or maybe seen alone [2-3].

In 2001, Pfirrmann et al. described a spinal disk degenerative scoring model based on the MR imaging findings deviated from the disk structures, which helps evaluate the degree and grading of degenerative changes on the intervertebral disk spaces [4].

Our aim was to evaluate the radiological findings of patients with incidental fusion, assessing whether there is a correlation between the degenerative disk changes with hypermobility and aging processes.

## Materials and methods

A total of 52 vertebra levels from 45 patients (42.2% male (n = 19) and 57.8% female (n = 26)) with a mean age of 43.60 ± 18.92 (range of 11-84) was retrospectively studied. The distribution of the fused vertebrae was evaluated as three separate levels of the cervical, thoracal, and lumbar regions (Table 1).

**Table 1:** Distribution of descriptive properties

Age (year) (n=45)	Min-max (median)	11–84 (45)
	Avrg±Ss	43.60±18.92
Gender (n=45)	Female	26 (57.8)
	Male	19 (42.2)
Zone (n=52)	Cervical	27 (51.9)
	Thoracal	17 (32.7)
	Lumbar	8 (15.4)
Fused level (n=52)	C2–C3	12 (23.1)
	C3–C4	5 (9.6)
	C4–C5	1 (1.9)
	C5–C6	4 (7.7)
	C6–C7	2 (3.8)
	C7–T1	3 (5.8)
	T1–T2	3 (5.8)
	T2–T3	2 (3.8)
	T3–T4	6 (11.5)
	T3–T4–T5	2 (3.8)
	T4–T5	2 (3.8)
	T7–T8	1 (1.9)
	T8–T9	1 (1.9)
	T12–L1	4 (7.7)
	T12–L1–L2	2 (3.8)
	L1–L2	1 (1.9)
	L4–L5	1 (1.9)

The inclusion criterion was incidental fusion, excluding other fusion causes. All patients’ MRI data were evaluated according to the Pfirrmann grading system, which was used to evaluate the segments of the upper and lower parts of the fused area separately. Also, the Pfirrmann grading system was calculated every three upper and lower levels from the fusion, finding a true correlation between the effects of hypermobility over the adjacent segments.

The examination was performed on a 3T whole-body magnetic resonance system (MAGNETOM Skyra 1.5T; Siemens Healthcare, Erlangen, Germany). The patients’ MRI data were divided into five groups based on the Pfirrmann grading system. The data were evaluated on each cervical, thoracal, and lumbar intervertebral disk level adjacent to the fused vertebrae and up to three levels above and below from the fusion.

Statistical reviews

The Number Cruncher Statistical System (NCSS) 2007 and the Power Analysis and Sample Size (PASS) 2008 statistical software (Utah, USA) were used for the statistical analysis. The Shapiro-Wilk test and the boxplot charts were used in the normal distribution of variables and descriptive statistical methods (mean, standard deviation, median, frequentness, ratio) upon study data evaluation. The Wilcoxon signed-rank test was used in the in-group evaluations of the Pfirrmann scores, which did not show a normal distribution. Meaningfulness was evaluated at p<0.05.

## Results

**Figure 1:**
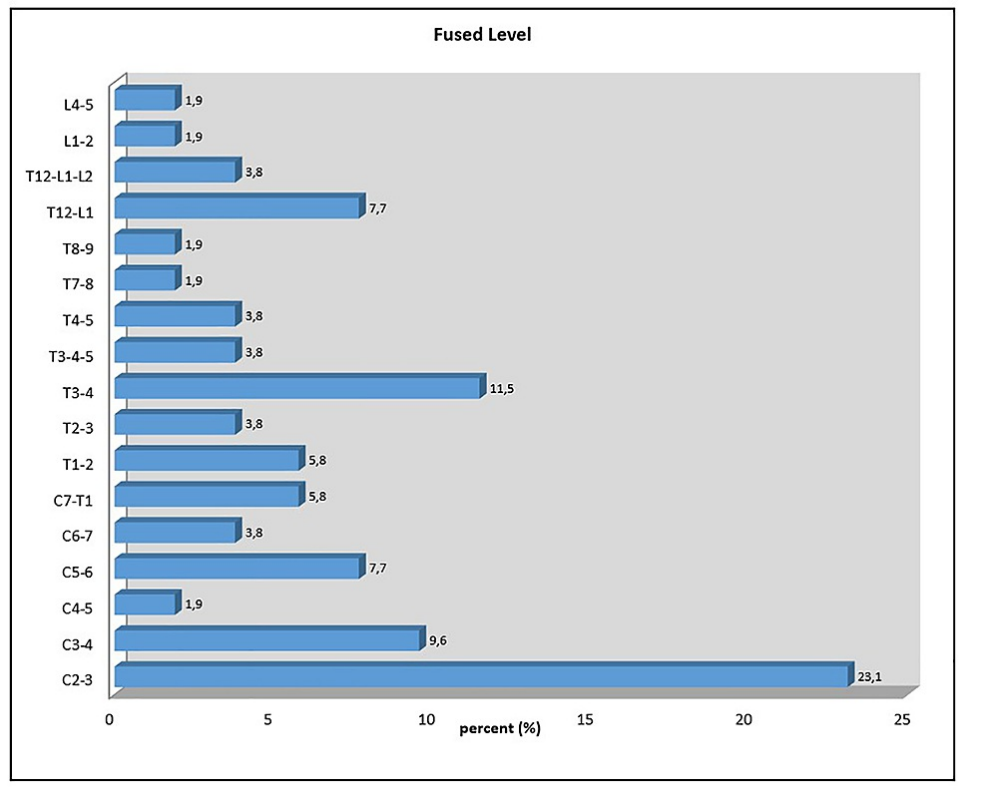
Fused level distributions

The highest rate of incidental fusion is determined on the cervical area with 51.9%, followed by the thoracal area with 32.7%, and the lumbar area with 15.4%. When the vertebra event was examined within the regions, a 23.1% C2-C3 vertebra merger was determined, followed by T3-T4 with 11.5% and C3-C4 with 9.6%. The distributions in other vertebrae are seen in Figure 1.

In the cervical area

The decrease in the segment measurements in the upper segment Pfirrmann scores at the first and second close was found to be statistically significant (p<0.01 and p<0.05, respectively). The decrease in segment measurements at the third close relativity and Pfirrmann mean scores (Pfirrmann 1, 2, and 3) according to the upper segment Pfirrmann scores was found to be statistically significant (p<0.05 and p<0.01, respectively).

The decrease in the segment measurements at the first and second close relativity according to the lower segment Pfirrmann scores was also statistically significant (p<0.01 and p<0.01, respectively). The decrease in the segment measurements at the third close relativity according to the upper segment Pfirrmann scores was again statistically significant (p<0.01).

**Figure 2:**
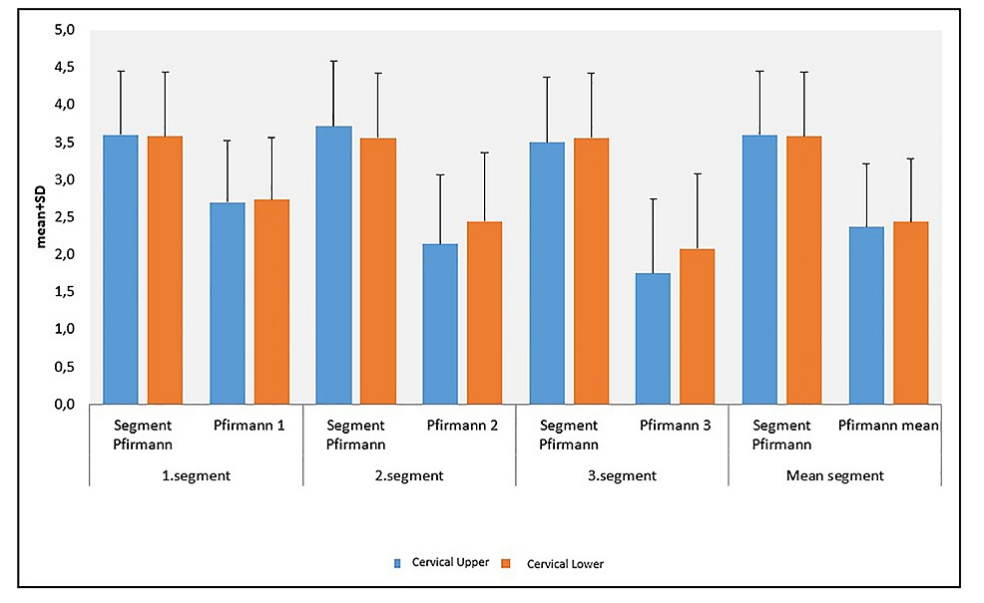
Distribution of the cervical’s upper and lower Pfirrmann measurements

According to the lower segment Pfirrmann scores, the decrease in Pfirrmann mean scores (Pfirrmann 1, 2, and 3) was also statistically significant (p<0.01) (Figure 2; Table 2).

**Table 2:** Distribution of the Pfirrmann scores in the upper, lower adjacent segments and the segments far from fused segments received by closeness

		Cervical			Thoracal			Lumbar		
		N	Median (Q1–Q3)	p	N	Median (Q1–Q3)	p	N	Median (Q1–Q3)	p
Upper	Segment Pfirrmann	10	4 (3–4)	0.003**	16	2 (1–3)	0.047*	7	2 (2–3)	0.157
	Pfirrmann 1	10	3 (2–3.3)		16	2 (1–2)		7	2 (2–2)	
	Segment Pfirrmann	4	4 (3–4)	0.015*	15	2 (1–3)	0.084	7	2 (2–3)	0.180
	Pfirrmann 2	4	2 (2–3)		15	2 (1–2)		7	2 (1–2)	
	Segment Pfirrmann	10	4 (3–4)	0.049*	16	2 (1–3)	0.212	7	2 (2–3)	0.102
	Pfirrmann 3	10	2.4 (1.8–3)		16	2 (1–2.6)		7	2 (1.3–2)	
	Segment Pfirrmann	10	4 (3–4)	0.004**	16	2 (1–3)	0.066	7	2 (2–3)	0.109
	Pfirrmann mean	10	2.4 (1.8–3)		16	2 (1–2.6)		7	2 (1.3–2)	
Lower	Segment Pfirrmann	26	4 (3–4)	0.001**	15	3 (1–4)	0.004**	7	3 (2–4)	0.046*
	Pfirrmann 1	26	3 (2–3)		15	2 (1–2)		7	2 (2–3)	
	Segment Pfirrmann	25	2 (2–3)	0.001**	15	3 (1–4)	0.007**	7	3 (2–4)	0.102
	Pfirrmann 2	25	2 (2–3)		15	2 (1–2)		7	3 (1–3)	
	Segment Pfirrmann	25	4 (3–4)	0.001**	14	3 (1–4)	0.007**	7	3 (2–4)	0.034*
	Pfirrmann 3	25	2 (1–3)		14	1.5 (1–2)		7	2 (1–2)	
	Segment Pfirrmann	26	4 (3–4)	0.001**	15	3 (1–4)	0.005**	7	3 (2–4)	0.044*
	Pfirrmann mean	26	2.5 (1.9–3)		15	2 (1–2)		7	2.3 (1.3–3)	

In the thoracal region

A slight decrease in the segment measurements at the first close relativity according to the upper segment Pfirrmann scores was found to be statistically significant (p<0.05). The changes seen in the second and third close segment measurements according to the upper segment Pfirrmann scores were not statistically significant (p>0.05). There was also no statistically significant change in the Pfirrmann average scores (Pfirrmann 1, 2, and 3) according to the upper segment Pfirrmann scores (p>0.05).

**Figure 3:**
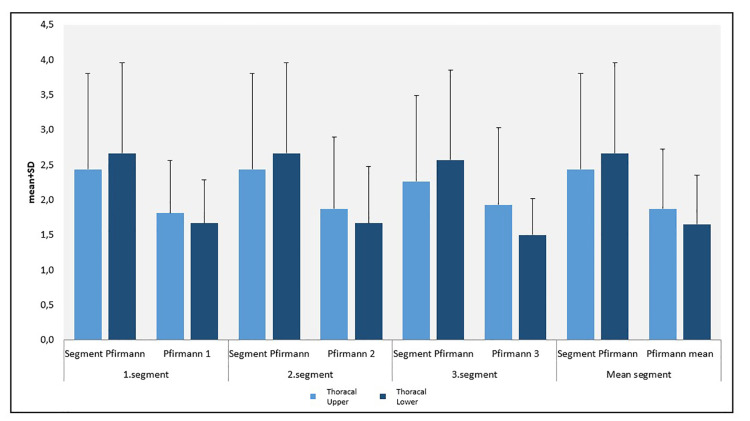
Distribution of the thoracal upper and lower Pfirrmann measurements

The decrease in the segment measurements at the first and second close relativity according to the lower segment Pfirrmann scores was also statistically significant (p<0.01 and p<0.01, respectively). The decrease in the segment measurements at the third close relativity and in the Pfirrmann mean scores (Pfirrmann 1, 2, and 3) according to the lower segment Pfirrmann scores were again statistically significant (p<0.01 and p<0.01, respectively) (Figure 3; Table 2).

In the lumbar region

The changes seen in the first, second, and third close segment measurements and the changes in the Pfirrmann average scores (Pfirrmann 1, 2, and 3) according to the upper segment Pfirrmann scores were not statistically significant (p>0.05 and p>0.05, respectively).

The decrease in the segment measurements at the first close relativity according to the lower segment Pfirrmann scores was also statistically significant (p<0.05). The lower segment Pfirrmann scores show no statistically significant change of the second close segment measurements (p>0.05). The decrease in the segment measurements at the third close relativity according to the upper segment Pfirrmann scores was found to be statistically significant (p<0.05).

**Figure 4:**
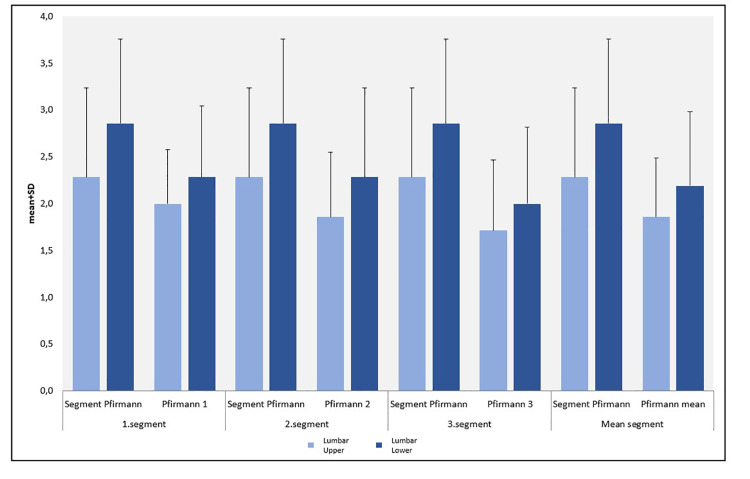
Distribution of the lumbar’s upper and lower Pfirrmann measurements

According to lower segment Pfirrmann scores, the decrease in the Pfirrmann mean scores (Pfirrmann 1, 2, and 3) was also statistically significant (p<0.05) (Figure 4; Table 2).

## Discussion

ASD is the most frequent complication in spinal surgery, and it is still a debatable topic. Previous reports ranging from 1.4% to 16.8% of reoperation needed ASD after the first operation [5]. The main inquiry is on whether there is a correlation between spinal fusion surgery and adjacent segment degeneration and if it is a natural process occurring with embryogenesis and aging.

Two important reasons exist for the emergence of the disease. The first one is hypermobility in the upper and lower segments close to the fusion. Increasing stresses on the upper and lower segments and following intradiscal-elevated pressure causes early degeneration [6]. The second one is the natural aging process [7]. Nowadays, there is no common opinion about the precise trigger factors of ASD.

Additional comorbid factors, such as aging, female gender, hormonal disproportions, preexisting degenerative disks adjacent to instrumentation, injury to facet joints adjacent to instrumentation, the right selection of instrumentation techniques, screw length and diameter, insertion degree, and the bi-cortical placement of screws, have an important and crucial role [8-9]. Attention to the spine’s normal crossing points to preserve the normal range of motion and prevent hypermobility is important. Considering the sagittal balance and the correction of malalignments peroperatively may prevent the consequent disproportionate distribution of loads. Also starting or ending the insertion of the instrumentation material at the transition points of the spine may increase the risk of hypermobility [5,10-11]. The presence of malalignment on the endpoint sites of instrumentation materials has been shown to possibly lead to ASD due to hypermobility [12].

Iatrogenic injury to the anatomical structures of functional spinal units (FSUs) during surgical procedures causes disproportionate load sharing on the upper and lower contiguous segments of the fused vertebra, which results in the disappearance of the biomechanical properties of the spine according to Punjabi’s three-column concepts, well-known to contribute to ASD [13].

However, there is no clear statistical data on the possibility of fusion surgeries to increase ASD incidence. Its frequency was reported to be between 5.6% and 30%. A seven-year follow-up study of short-segment fusion surgery had shown around a 25% subsequent ASD incidence, where 5.6% of the cases required additional operation [14]. There is a correlation between ASD incidence and the elongation of fusion systems over the three segments, which may be due to overloading on adjacent disks and hypermobility [9,15-18].

Biomechanical studies demonstrate the fact that as the mobility of a segment increases, the incidence of ASD development increases parallelly [13]. Dynamic instrumentation systems may preserve FSU mobility and range of motion (ROM), which may help reduce the adjacent segments’ hypermobility, reducing the occurrence rate of degenerative processes. Some clinical trials claimed that it not only preserves the adjacent segments’ normal range of motion but also proportionates the distribution of axial loads [19-20]. However, it needs more clinical testing and comparisons of the long-term postoperative results of both rigid and dynamic instrumentation systems [21-22].

In our study, we found significant decreases in the mean Pfirrmann scores of the segments far from the fusion when compared to the segments close to the fusion with the highest rate of degeneration. It showed that the disks adjacent to the fusion are the most affected area by hypermobility, especially in the cervical region. Also, previously carried out studies have shown that hypermobility syndrome accelerates the adjacent segments’ degeneration rate [23-25]. The same result was found in the lower segments of the lumbar area, which also supported the same hypothesis. The result of mean Pfirrmann in the thoracal and upper lumbar regions was not statistically remarkable. In our opinion, it depends on the fact that the thoracal region is the less mobile part of the spine. So, the effect of hypermobility syndrome on the disks will not be significant. It may also clarify the meaningless result in the P-values of the upper lumbar mean Pfirrmann grading that is probably caused by the overlapping of the mean Pfirrmann results on the thoracal region.

However, the degree of disk degeneration in older patients was more remarkable than those in younger patients. It may not directly depend on aging processes. That may more likely be due to the fact that hypermobility worsens by aging. Conversely, this is exactly the reason that disk degeneration increases with aging.

## Conclusions

ASD may be influenced by factors beyond age. The use of fusion instrumentation systems could trigger some degenerative processes due to hypermobility over time, i.e., hypermobility initiates a number of pathophysiological processes, causing progressive deterioration of adjacent segments over time. Our findings suggest that beyond physiological aging, this is likely an event mediated by hypermobility over time. Further studies should be done to better understand this issue with a larger cohort of patients
